# The Coevolution of Phycobilisomes: Molecular Structure Adapting to Functional Evolution

**DOI:** 10.1155/2011/230236

**Published:** 2011-08-29

**Authors:** Fei Shi, Song Qin, Yin-Chu Wang

**Affiliations:** ^1^The Coastal Zone Bio-Resource Laboratory, Yantai Institute of Coastal Zone Research, Chinese Academy of Sciences, Yantai 264003, China; ^2^Yantai Institute of Coastal Zone Research, Graduate University of the Chinese Academy of Sciences, Beijing 100049, China

## Abstract

Phycobilisome is the major light-harvesting complex in cyanobacteria and red alga. It consists of phycobiliproteins and their associated linker peptides which play key role in absorption and unidirectional transfer of light energy and the stability of the whole complex system, respectively. Former researches on the evolution among PBPs and linker peptides had mainly focused on the phylogenetic analysis and selective evolution. Coevolution is the change that the conformation of one residue is interrupted by mutation and a compensatory change selected for in its interacting partner. Here, coevolutionary analysis of allophycocyanin, phycocyanin, and phycoerythrin and covariation analysis of linker peptides were performed. Coevolution analyses reveal that these sites are significantly correlated, showing strong evidence of the functional and structural importance of interactions among these residues. According to interprotein coevolution analysis, less interaction was found between PBPs and linker peptides. Our results also revealed the correlations between the coevolution and adaptive selection in PBS were not directly related, but probably demonstrated by the sites coupled under physical-chemical interactions.

## 1. Introduction

The process of photosynthesis is initiated by the absorption of light. In cyanobacteria and red algae, the main accessory light-harvesting complexes are comprised of the phycobilisomes (PBSs), which are attached to the cytoplasmic surface of the thylakoid membrane except *Gloeobacter violaceus *PCC7421 having no thylakoid membrane [1–6]. PBSs are composed of rods and a core and biochemically consist of phycobiliproteins (PBPs) and linker polypeptides, which are particularly superior subjects for the detailed analysis of structure and function due to their various components affected by growth conditions [2]. In view of the spectral properties as well as pigment compositions, allophycocyanin (APC), phycocyanin (PC), and phycoerythrin (PE) are the principal classes of PBPs in cyanobacteria. They consist of two different subunits, *α* and *β*, which exhibit high affinity for one another and associate into (*α*/*β*)-monomers to be organized as (*α*/*β*)_3_-trimers and (*α*/*β*)_6_-hexamers [7]. Different PBPs contain different kinds and different numbers of chromophores, covalently attached to the apoprotein by thioether bonds to cysteine residues. PC has three phycocyanobilin chromophores attached to the monomer through thioester linkages at the *α*84, *β*84, and *β*155 positions [8, 9]. In addition, unlike PBPs, most of the linker polypeptides do not bear chromophores [10]. Previous studies have provided a system of abbreviations to characterize linker peptides in PBSs: rod linker (L_R_), rod-core linker (L_RC_), core linker (L_C_), and core-membrane linker (L_CM_) [11, 12]. They can induce the aggregation of the PBP trimers (L_R_) and also connect the rods to the core (L_RC_), and the core to the thylakoid membrane (L_CM_). The light energy absorbed by PE is transferred to PC, and then to APC, finally to the chlorophyll a in a quite efficient way [2, 4]. PBPs are important for absorbing light energy, while the linker polypeptides are important for stability and assembly of the complex.

Previous researches are mainly focused on PBPs. Electron microscopic and crystallographic studies have revealed that the tertiary fold and the general architecture of macromolecular assemblages are remarkably conserved and provided a wealth of information on structure and function relationship of PBPs [13–18]. Amino acid sequence alignments and phylogenetic analyses have been used to go through the parse for the evolution of PBPs [7]. Also, the divergence and evolution of linker family have been investigated [19].

 Light quality and quantity are key factors affecting the composition of PBSs. Two different forms of PE gene, found in two ecotypes of *Prochlorococcus*, are specifically adapted to either high-light (HL) or low light (LL) conditions which are under different selective pressure [19–21]. The structure and function of linker peptides in PBSs have shown a great diversity based on the light condition [1, 22]. The method to respond to high-light stress in marine cyanobacteria is decreasing the content of PBSs per cell [23]. 

 As we know, coevolution is prevalent at species as well as molecular levels. In the molecular level, coevolution between amino acid sites can be the result of their structural, functional, physical interaction, phylogenetic convergence, and their stochastic covariation [24]. Coevolving sites are a powerful indicator of the structures, interactions, and functions between residues [25–27]. The strength and pattern of coevolution vary depending on their environment. Since the nature and strength of residue interactions vary according to the involved residues and their local and global environments, coevolution exhibits a complex dependence [28].

The availability of protein sequences and their previous information allow us to perform a systematic screening on PBS protein families. Here we extended an exhaustive coevolution analysis of PBP genes and the linker polypeptide genes from the well-annotated and even unfinished cyanobacterium and red alga genomes. Intramolecular and interprotein coevolution of PBPs and covariation analysis of linker peptides in the varieties of PBSs were analyzed, and specific comparison to positive selection was also performed for better understanding the evolution of PBSs.

## 2. Materials and Methods

### 2.1. Sequence Collection, Alignment

For the large amounts of data, sequences with PBPs and linker peptides in 21 cyanobacteria and 5 red algae were obtained from GenBank with the accession numbers which could be found in the accessory files. The 21 cyanobacteria and 5 red alga are *Synechocystis *sp. PCC 6803, *Nostoc* sp. PCC 7120,* Microcystis aeruginosa *NIES-843, *Cyanothece *sp. ATCC 51142, *Gloeobacter violaceus *PCC 7421, *Synechococcus *sp. JA-2-3B′a(2–13), *Nostoc punctiforme *PCC 73102, *Synechococcus *sp. JA-3-3Ab, *Synechococcus elongatus *PCC 6301, *Synechococcus *sp. PCC 7002, *Synechococcus *sp. WH 8102, *Thermosynechococcus elongatus *BP-1, *Arthrospira platensis* str. Paraca, *Synechococcus* sp. CC9902, *Synechococcus *sp. CC9605, *Synechococcus *sp. CC9311, *Cyanothece *sp. PCC 7424, *Cyanothece *sp. PCC 7425, *Cyanothece *sp. PCC 8801, *Lyngbya *sp. PCC 8106, *Arthrospira platensis*, *Porphyra yezoensis*,* Cyanidium caldarium*, *Porphyra purpurea*, *Cyanidioschyzon merolae *strain 10D, and *Gracilaria tenuistipitata *var.* liui*. Amino acid alignments were carried out using CLUSTAL X [29, 30] and MUSCLE [31] software and then manually adjusted using BioEdit (http://www.mbio.ncsu.edu/BioEdit/bioedit.html). Further analyses were all performed on this set of aligned amino acid sequences.

### 2.2. Coevolution Analysis

Many methods, parametric or nonparametric, suffer from inaccuracies from their inability to erase the background noise [24, 25]. Coevolution analysis using protein sequences (CAPS) compares the correlated variance of the evolutionary rates corrected by the time based on the divergence of the protein sequences [25]. It uses the blocks substitution matrix (BLOSUM) method between two sequences at these particular sites [32]. This application is based on CAPS Version 1.0 [33]. This method has proved to be successful in disentangling real coevolutionary signal from the background noise and minimizing false positive rate with high sensitivity [25]. CAPS can produce the files containing information of coevolutionary networks and compensatory mutations. CAPS program is available at http://bioinf.gen.tcd.ie/caps/. Also, we run the InterMap 3D 1.3 server (http://www.cbs.dtu.dk/services/InterMap3D/) [34, 35] to measure the atomic distance as a complementary explanation for the evidence of coevolution. 

### 2.3. Covariation Analysis

Detecting structural interactions and statistical covariance among separate amino acid sites is significant for understanding protein covariation and evolution [27, 36]. Such analyses are based on the assumption that functionally significant coordinated residues in proteins originated by physicochemical properties (e.g., volume, charge, polarity, and hydrophobicity) of the residues [37]. Here, we use the software CRASP (Correlation analysis of the amino acid substitutions in protein sequences) to run the coevolved analysis. The CRASP program is available at http://wwwmgs.bionet.nsc.ru/mgs/programs/crasp/. 

## 3. Results

### 3.1. Intramolecule Coevolution Analysis

**Figure 1:**
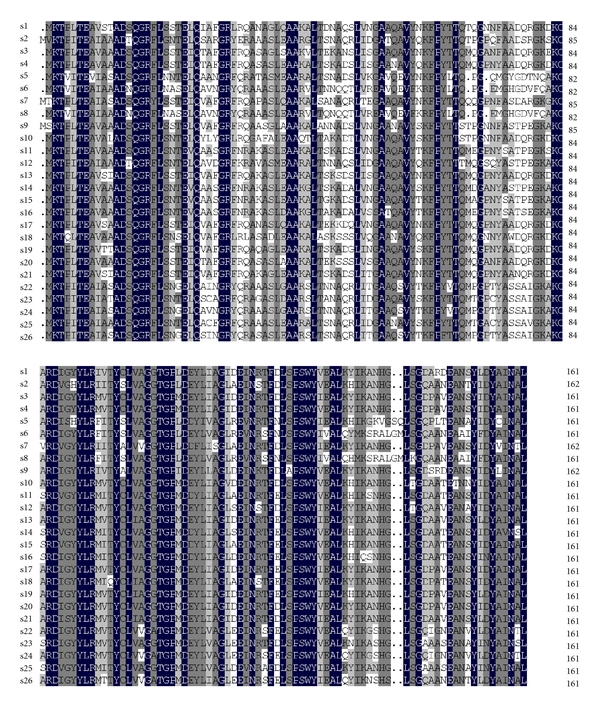
Multiple sequence alignments of PC *α* subunits in cyanobacteria and red algae. PC-*α* (species name, accession number): S1:* Synechocystis sp. *PCC 6803, NP_440551.1; S2:* N. sp. *PCC 7120, NP_484573.1; S3: *M. aeruginosa *NIES-843, YP_001657460.1; S4:* C. sp. *ATCC 51142, YP_001804066.1; S5: *G. violaceus *PCC 7421, NP_924131.1; S6: *Synechococcus sp. *JA-2-3B′a(2–13), YP_477182.1; S7: *N. punctiforme *PCC 73102, YP_001868554.1; S8: *S. sp. *JA-3-3Ab, YP_473707.1; S9: *S. elongatus *PCC 6301, YP_171205.1; S10: *S. sp. *PCC 7002, YP_001735446.1; S11: *S. sp. *WH 8102, NP_898114.1; S12: *T. elongates *BP-1, NP_682748.1; S13: *A. platensis str. Paraca,* ZP_06380686.1; S14: *S. sp. *CC9902, YP_377910.1; S15: *S. sp. *CC9605, YP_380751.1; S16: *S. sp. *CC9311, YP_729715.1; S17: *C. sp. *PCC 7424, YP_002375498.1; S18: *C. sp. *PCC 7425, YP_002482426.1; S19:* L. sp. *PCC 8106, ZP_01619119.1; S20: *C.sp. *PCC 8801, YP_002373212.1; S21: *A. platensis*, ABD64608.1; S22: *P. yezoensis*, YP_537059.1; S23: *C. caldarium*, NP_045082.1; S24: *P. purpurea*, NP_053988.1; S25: *C. merolae strain 10D*, NP_848986.1; S26: *G. tenuistipitata var. liui*, YP_063694.1.

**Figure 2:**
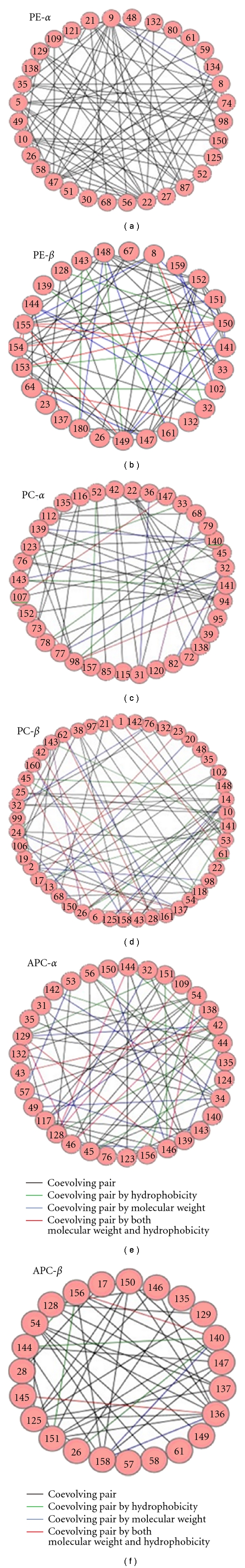
Intramolecular coevolutionary networks in *α* and *β* subunits in PBPs. Group-specific coevolutionary networks for PE, PC, and APC *α* and *β* subunits are shown. Sites under potential coevolution efforts are identified using *S.* sp. PCC 6803 sequences in APC and PC, *S.* sp. WH8102 in PE as the references. Nodes for amino acid sites are connected through edges colored according to the characteristics of mutation coevolutions.

Figure 1 shows amino acid sequences alignment of PC *α* subunits. In addition, numbers of highly conserved amino acids of the PBPs were identified from the sequence alignments (see detailed information in the supplementary materials available online at doi: 10.1155/2011/230236).  Figure 2 provides clear coevolution relevance with *α* and *β* subunits in PE, PC, and APC, respectively. In addition to the implementation of the method previously published [25], CAPS also performs a preliminary analysis of compensatory mutations by testing the correlation in the hydrophobicity and the molecular weight variations between coevolving amino acids [33]. Some of the coevolving groups detected are significantly correlated either in hydrophobicity or molecular weight or both (details are shown in Table 1). 

PC and APC are common in cyanobacteria and red alga, while PE just exists in less species. In PE *α* subunit, few physicochemical properties among coevolved amino acid residues with no groups in hydrophobicities were detected. Just one coevolved pair (V8 and V9) were detected correlation in molecular weight with *ρ* = 0.9159 and *P* = 0.0036 showed high robustness.

### 3.2. Interprotein Coevolution Analysis

Interprotein coevolution, in addition to the intramolecular analysis developed previously, can also be operated by CAPS. Detecting correlation in the molecular weights and hydrophobicities in the groups of coevolution is not available in such condition.

**Figure 3:**
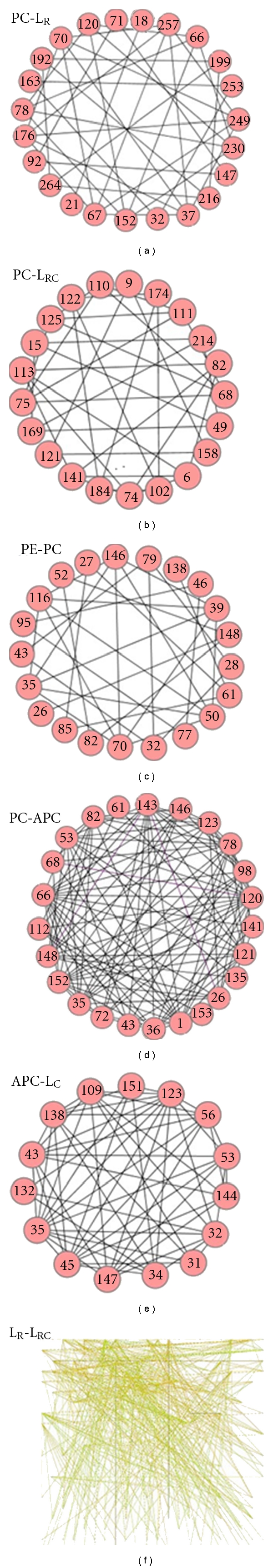
Interprotein Coevolutionary networks in PBSs. Six interprotein coevolutionary networks PC-L_R_, PC-L_RC_, PE-PC, PC-APC, APC-L_C_, and L_R_-L_RC_ are shown. Nodes for amino acid sites are connected through edges colored according to the characteristics of mutation coevolutions. The event L_R_-L_RC_ with numerous coevolutionary residues is shown by this two-dimensional chart.

We run all the possible interprotein coevolution analysis according to the locations in PBSs, including two proteins of PBPs or linker peptides or both. The linker peptides had few connections to PBPs according to their coevolution results. No coevolution groups were found in the CAPS output in APC-L_CM_, L_C_-L_CM_, PE-L_R_, and L_RC_-L_C_.  Figure 3 shows six interprotein coevolution networks in PC-L_R_, PC-L_RC_, PC-APC, PE-PC, APC-L_C_, and L_R_-L_RC_. Compared to intramolecular coevolution, less groups were found in interprotein analysis. Besides, it is obvious that the relationships among two proteins of PBPs or linker peptides were much closer than the connections between the PBPs and linker peptides. 

### 3.3. Covariation Analysis

**Figure 4:**
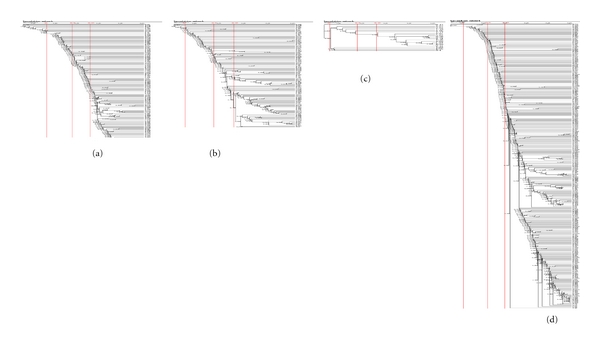
Linker peptides L_R_, L_RC_, L_C_, and L_CM_ (a–d) correlation networks of covariation analysis. The number below each node indicates the correlation coefficient value. The vertical gray bars indicate different significance thresholds.

These characteristics of amino acids reflect physical and chemical interactions between residues. It has been suggested that these linker proteins play roles in rod-core assembly and complex stabilization [38]. There are many physical-chemical scale parameters such as flexibility, volume, polarity, and hydrophobicity. Here, we firstly considered such amino acids characteristics as volumes for covariation analysis. As can be seen in Figure 4, nearly all the lineages were highly correlated at the 99% significance level, while some of them approaching to 99.99%. In L_C_, the lengths of the sequences are very short (approximately 67 amino acids); thus the number of the coevolved sites were the least. Other linker peptides contain numerous coevolved residues owning to the physical-chemical interactions. Then alignment consequences of these peptides are narrowly conservative. The number of amino acids, residue-residue interactions, the dependence of covariations on phylogenetic distances and interior environment would be the main factors to account for the covariation outcomes. Then we chose some other amino acid characteristics (polarity, hydrophobicity, and flexibility) to perform the covariation analyses. Results from the properties are similar to the former analysis, just changing the branch locations with the same residues. 

### 3.4. Atomic Distance

The analysis of the atomic distances (AD) identified a certain percentage of coevolving residues within each group as spatially close. Physical distance (<10 Å) is one pattern within the residues in the coevolutionary events [39]. 

**Figure 5:**
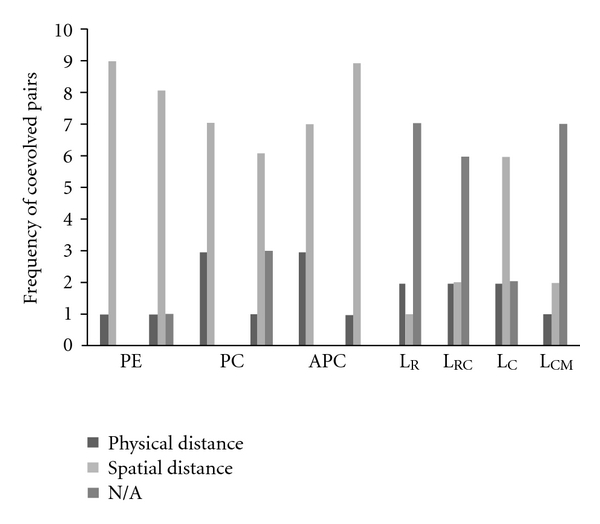
The distribution of the atomic distances in PBSs. Frequencies of coevolved functionally important pairs are plotted versus *α* and *β* subunits in PBPs and linker family. Bars represent frequencies of atomic distances within physical distance, spatial distance, and not available, respectively.

In Figure 5, large amounts of spatial couplings and few physical interactions were detected in all PBPs and linker peptides.

 Spatially proximal pairs of sites and clusters of distant sites located in functional domains, suggest a functional dependency between them [39]. Furthermore, linker family shares the coevolution positions in which most atomic distances showed not available for their unknown protein tertiary structures. 

## 4. Discussion

Intramolecular coevolution detected among PBPs reveals the strong coevolved connections between sites. The factors on compensatory mutations including hydrophobicities and molecular weights are among the most important in explaining amino acid contribution to protein structure with less error [40]. Most of the coevolving residues are significantly correlated in hydrophobicities and molecular weights except the PE *α* subunit. It may be caused by the microenvironment of PBS. The interactions of hydrophobicities are responsible for different phenomena such as structure stabilization of proteins [41] and folding of proteins [42]. PE is the outmost portion of the structure of PBS, so it might possess less physical-chemical interactions than APC and PC. Other possible explanations include the coupling patterns which can balance the formation of the region and the interior environment such as water dynamics. 

Apt proposed a hypothetical outline that different types of PBPs and linker peptides originated from the same ancestor [7]. The results of interprotein coevolution analysis in PBPs verify the previous hypothesis, and so as linker peptides. Apt and Zhao also supposed that the linker polypeptides developed from an earlier ancestor of PBPs [7, 43]. The rare relationship between PBPs and linker peptides depending on the interprotein coevolution analysis demonstrates less interaction in the long period of evolution. This hypothesis would in part be overturned by this point. 

Interestingly, a significant proportion of the sites detected coevolving had been previously proposed to be under adaptive evolution [25, 39]. Based on* S. *sp. PCC 6803 PC-*α* protein numbering in Zhao's research [43], these residues are 4P, 5L, 7E, 15Q, 25Q, 66T, 88I, 107L, 118S, 119P, 134K, and 140H (these positions are not the same with the paper [43] for they edited the sequences) under positive selection with posterior probability >0.95. Only two positions 107L and 140H are involved in the coevolutionary analysis. The positive selective residues are usually between or adjacent to the coevolutionary sites, such as 118S and 119P within the coevolved residues 115I, 116D, and 120R. In PC-*β* subunit, 30T, 57R, 61A, 103S, 127V, 129A, 130G, 133K, 139L, 167A, 168A, and 171V were under the adaptive selection. Surprisingly, just one residue 61A was detected coevolved. Most of the sites are also among the coevolved sites that shows the potential connection between coevolved and selective residues. And we found that the coevolution positions occurred through the whole molecule, while many sites with elevated dN/dS ratios (the frequency of nonsynonymous versus synonymous substitutions) in different PBP lineages were located in the chromophore-binding domain and the helical hairpin domains (X and Y) [43]. 

The identification of genes showing particular amino acid residues that have undergone adaptive evolution is a key in determining functionally or structurally important protein regions. Conserved amino acids throughout protein evolution are expected to have critical effects on protein functions [44, 45]. Former researches had concluded the relationship between coevolution and selective pressure with the fixed associations [25, 43]. In this paper, we found that most of the sites under adaptive selection were adjacent to or among the coevolved ones. One hypothesis is that according to the physical-chemical properties, the residues under positive selection are one key factor to stimulate the sites coevolved and *vice versa*. Coevolutionary sites may be important in two ways. First, some sites are functionally important because they provide the ability to respond to the dynamic circumstance [46, 47]. Second, the regions may be indirectly important because they fall in the vicinity of important amino acid sites, and therefore their variability may dramatically affect functional sites. In the latter case, variable amino acid sites tend to coevolve to preserve the structural characteristics of the functional sites [26]. It is expected that compensatory coevolution may occur either between amino acid sites three dimensionally proximal (indicating structural and probably functional coevolution) or alternatively between sites apparently far apart from one another but in contact with functionally important sites. Certain variability coupled with the strong functional constraints and the involvement in the network of interactions for coevolutionary processes would both arise from the environmental factors especially light acclimation. Hence, the complex relations between coevolution and selective constraints are worth pursuing at a deeper level.

The coevolutionary analysis is regarded as an important tool to gain functional and structural relationships in a protein. The evolution of amino acid residues is hence depending on their mutation and the constraint pressure imposed by their complex networks [48]. Amino acid interdependency can lead to coevolution. Many evidences pointed to the importance of coevolution in shaping the molecular function [24, 25, 27]. Moreover, structural and functional coupling of distant interacting residues requires coevolution among these amino acid residues. Some possible explanations include the coupling of binding energy via pathways in the protein, interactions with intermediate molecules, and the surrounding environment. Various environmental factors especially light acclimation were the primary influences in coevolution. And the detail evolution mechanism in PBSs mediating by the light can be further resolved. 

## Supplementary Material

(1) The species and accession numbers of PBS
Ranked correlation coefficient for coevolving amino acid sites in PBPs. Plots of correlated variations in hydrophobility (A–E) and molecular weight (F–J) among amino acid sites for PE-**β** subunit, PC and APC **α** and **β** subunits, respectively. Less complementary mutation coevolution was found in PE-**α** subunit.

## Figures and Tables

**Table 1:** The number of coevolving groups under different correlated types in PBPs.

Coevolution type	Number of coevolving groups					
	PE-*α*	PE-*β*	PC-*α*	PC-*β*	APC-*α*	APC-*β*
Coevolved groups	20	26	30	40	34	18
Hydrophobicity	0	14	9	18	12	3
Molecular weight	1	18	7	20	15	3
Hydrophobicity and molecular weight	0	9	4	15	7	1
