# Supplementary material for: The Coevolution of Phycobilisomes: Molecular Structure Adapting to Functional Evolution
Source: Comp Funct Genomics. 2011 Aug 29;2011:230236. doi: 10.1155/2011/230236 (PMC3166575; doi:10.1155/2011/230236)
Supplement: Supplementary file 1 — (1) The species and accession numbers of PBS Ranked correlation coefficient for coevolving amino acid sites in PBPs. Plots of correlated variations in hydrophobility (A–E) and molecular weight (F–J) among amino acid sites for PE-β subunit, PC and APC α and β subunits, respectively. Less complementary mutation coevolution was found in PE-α subunit. [file 230236.f1.docx]

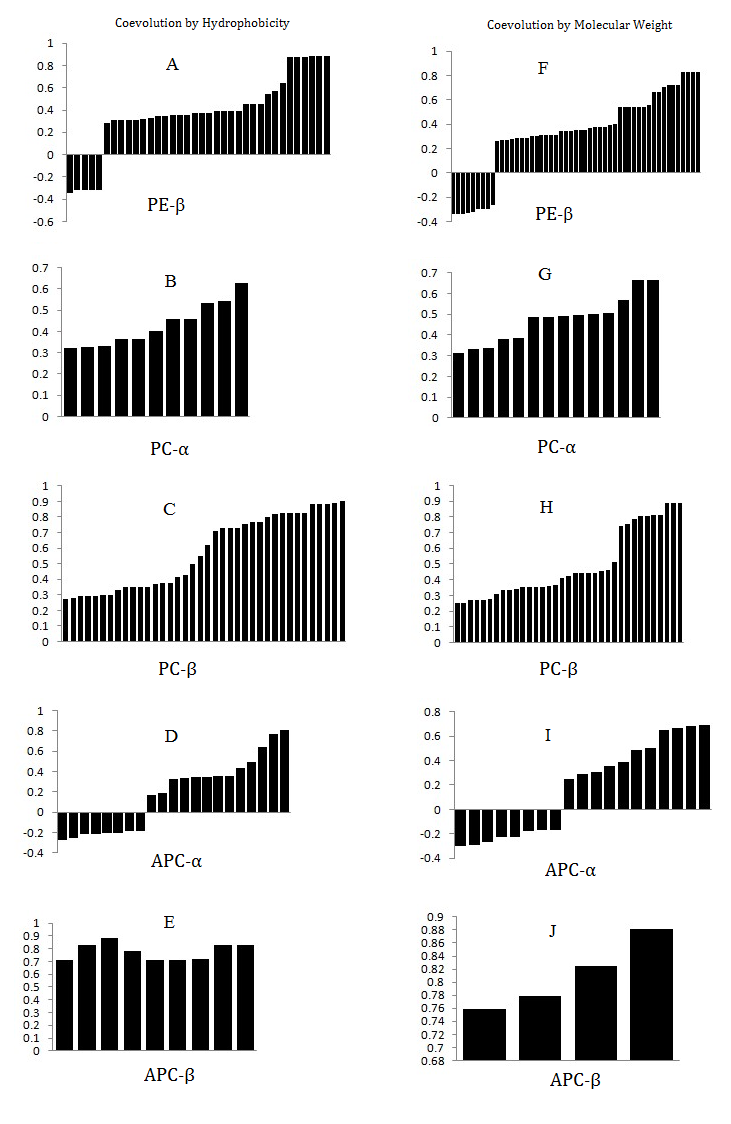


Figure 1. Ranked correlation coefficient for coevolving amino acid sites in PBPs. Plots of correlated variations in hydrophobility (A-E) and molecular weight (F-J) among amino acid sites for PE-β subunit, PC and APC α and β subunits, respectively. Less complementary mutation coevolution was found in PE-α subunit.
